# Deriving comprehensive literature trends on multi-omics analysis studies in autism spectrum disorder using literature mining pipeline

**DOI:** 10.3389/fnins.2024.1400412

**Published:** 2024-11-12

**Authors:** Dattatray Mongad, Indhupriya Subramanian, Anamika Krishanpal

**Affiliations:** Life Sciences Research, CTO Unit, Persistent Systems, Pune, India

**Keywords:** classification, summarization, topic modeling, generative AI, NER

## Abstract

Autism spectrum disorder (ASD) is characterized by highly heterogenous abnormalities in functional brain connectivity affecting social behavior. There is a significant progress in understanding the molecular and genetic basis of ASD in the last decade using multi-omics approach. Mining this large volume of biomedical literature for insights requires considerable amount of manual intervention for curation. Machine learning and artificial intelligence fields are advancing toward simplifying data mining from unstructured text data. Here, we demonstrate our literature mining pipeline to accelerate data to insights. Using topic modeling and generative AI techniques, we present a pipeline that can classify scientific literature into thematic clusters and can help in a wide array of applications such as knowledgebase creation, conversational virtual assistant, and summarization. Employing our pipeline, we explored the ASD literature, specifically around multi-omics studies to understand the molecular interplay underlying autism brain.

## 1 Introduction

Autism spectrum disorder (ASD) refers to a group of complex neurodevelopment disorders caused by differences in the brain that affect communication and behavior. ASD is often difficult to diagnose due to its complex and heterogenous etiology related to neurological development in interrelated systems. Individuals with ASD have an impact in their social, developmental, linguistic, and cognitive functions that evolve throughout their lifetime (Hus and Segal, 2021). It is crucial to characterize such a disorder and investigate the multiple layers of association to get the wholistic picture that can aid in treatment and betterment of affected individuals. Although there are standards such as DSM-5 scale and Developmental Diagnostic Dimensional Interview (3Di) that help in assessing and identifying the affected levels, there is still a lot of scope to further the assessment and treatment options (Kipkemoi et al., 2024). More genetic, social, and behavioral studies are in progress to improve early detection and intervention for ASD.

Multi-omics studies, integrating data from multiple high-throughput methods such as genomics, transcriptomics, proteomics, and epigenomics, have the potential to gain insights into complex molecular and genetic mechanisms that contribute to development of ASD (Higdon et al., 2015). Multi-omics studies have the potential to identify molecular subtypes and unravel novel targets and actionable biomolecules that can aid in the treatment and care for ASD patients (Higdon et al., 2015). Recent advances in sequencing technologies have enabled the identification of copy number variations (CNVs) and rare single nucleotide variants (SNVs) associated with ASD (Bacchelli et al., 2020; Fu et al., 2022; Wang et al., 2022; Zhou et al., 2022). As per latest release (release_16_01_2024) of ASD database, Simons Foundation Autism Research Initiative (SFARI), SNVs, and CNVs from 1,162 genes have been reported as genetic risk factors (Abrahams et al., 2013). As a result of increasing in multi-omics studies, a vast amount of data and literature has accumulated on platforms such as PubMed.

PubMed is the largest repository of published peer-reviewed scientific literature and acts as the gateway for directed search with its advanced query system. However, for a given query even with advanced and special filters, PubMed yields many results that involves huge amount of manual curation to identify relevant articles and further explore them for insights. Autism spectrum disorder (ASD), being an actively researched topic with multiple dimensions such as neuroscience, behavioral studies, diagnosis, and molecular mechanisms, had approximately 5,000 scientific publications in the last 1 year. Extracting meaningful clinical insights and knowledge from these large number publications is challenging due to data complexity, data volume, heterogeneity of ASD, and interdisciplinary nature of research.

Herein, we focus on analyzing the peer-reviewed scientific literature for identifying the trends and insights on multi-omics studies in ASD patients. We have designed a pipeline that can assist in mining large textual data such as biomedical literature for expediting extraction of relevant information. Leveraging topic modeling techniques and large language models (LLMs), the pipeline helps in simplifying text data by clustering them into thematic clusters. Topic modeling uses unsupervised methods to discover hidden patterns from a large collection of textual data (Barde and Bainwad, 2017). This machine learning technique analyzes textual data for similarity patterns and determines word groups that best represent a set of documents to create thematic clusters. We used topic modeling on PubMed abstracts to cluster articles with semantically related keywords and help in identifying the various keywords (topics) associated with the search query. Topic modeling can help in trend analysis and market survey and identify gap areas in research studies thus providing useful insights. Furthermore, we leverage LLMs to showcase possibilities of building an interactive Q&A and summarization model that can be of great value in scientific reporting.

## 2 Materials and methods

### 2.1 Data collection

In brief, a search was carried out with the query “(Autism Spectrum Disorder AND *Homo sapiens*) AND ((‘2013/01/01’[Date - Completion]: ‘3000’[Date - Completion]))” using *esearch*. A total of 28,304 abstracts published in last 10 years (as on 15 November 2023) were downloaded from PubMed. The abstracts were downloaded and extracted using a Biopython (Cock et al., 2009) implemented in a custom python script.

### 2.2 Topic modeling

Topic modeling using BERT embeddings and class-based Term Frequency–Inverse Document Frequency (c-TF-IDF) was performed as implemented in BERTopic library (v0.15.0) (Grootendorst, 2022). Although there are multiple methods such as LDA, NMF, and Top2Vec, BERTopic was chosen for its flexibility and user friendliness. Based on the analysis presented by Egger and Yu (2022), BERTopic showed the potency to extract useful information from unstructured textual data.

The abstract text was subjected to lemmatization and filtration of pronouns, determiners, and conjunctions using WordNetLemmatizer implemented in NLTK (3.8.1). Filtered abstracts were fitted on BERTopic model with different combinations of UMAP and HDBSCAN parameters, and seed topics were provided for guided modeling (Table 1). Final model was selected based on the model evaluation metric, topic coherence (C_v and C_umass) (Mifrah, 2020). This evaluation method can be defined as the degree of significance between the words inside a topic and its ease of interpretation from human perspective. While C_v helps in measuring the coherence or similarity of the documents within a topic, C_umass considers the document co-occurrence counts. Higher C-v and lower C_umass (closer to 0) help in choosing a good topic model.

**Table 1:** List of keywords used as seed list for guided topic modeling.

Seed list 1	Multi-omics	Pan-omics	Omics	Integrative omics	Multiple omics
Seed list 2	Genomics	Mutation	SNP	SNV	Genome
Seed list 3	Transcriptomics	RNA	Gene expression	Mirna	Transcriptome
Seed list 4	Epigenomics	Methylation	Methylome	Epigenetics	Epigenome
Seed list 5	Copy number alteration	Amplification	Deletion	Loss	Gain
Seed list 6	Metabolomics	Metabolome	Metabolite	Lipids	Metabolism
Seed list 7	Proteomics	Protein	Proteome	Biomarkers	Protein folding

### 2.3 Named entity recognition

The biological entities within each abstract text were predicted using HunFlair model (Weber et al., 2021) implemented in Flair NLP framework (v0.13.0). It recognizes five important biomedical entity types with high accuracy, namely *Cell Lines*, *Chemicals*, *Diseases*, *Genes,* and *Species*. The gene names and gene symbols predicted by HunFlair were cleaned and compared with gene symbols available in org.Hs.eg.db (v3.16.0) (Carlson, 2022) and cleaned manually.

### 2.4 Conversational Q&A and summarization using generative AI

We used GPT3.5-turbo model from Azure OpenAI to create Retrieval-Augmented Generation (RAG)-based conversational chat assistant to perform Q&A on the articles (free full-text articles and abstracts). We also used Google’s Gemini model from Google Cloud’s VertexAI to generate summarized content for selected topics.

### 2.5 Code availability

A Jupyter notebook containing all codes for PubMed abstract download, processing, topic modeling, and creating a Q&A model is submitted to GitHub at https://github.com/pslomics/ASD_multiomics_analysis.

## 3 Results

### 3.1 Literature mining pipeline

**Figure 1:**
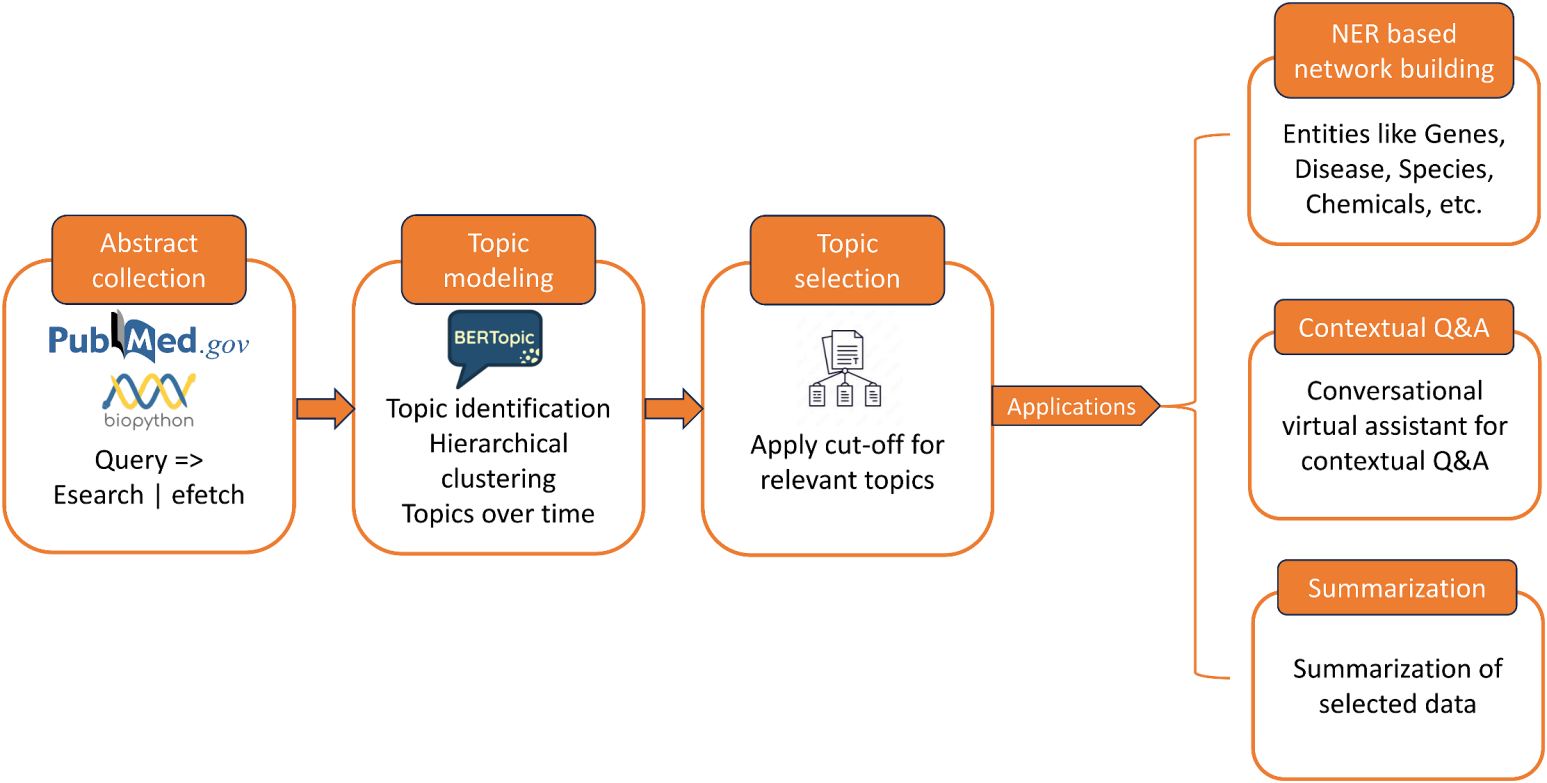
Overview of our literature mining pipeline and applications.

The pipeline is designed to simplify deriving insights from large, voluminous, and unstructured biomedical literature that is usually resource and time intensive. The pipeline focuses on grouping the articles into thematic clusters based on the representative words occurring in the abstracts. This helps in providing an overview of the different topics studied for a given query, and the representative topics can help in narrowing down the search space. Thus, the articles are clustered into different topics, and specific topics of interest can be shortlisted for further study. Upon selection of topics, a wide array of applications can be automated to achieve meaningful insights from the data. We showcase that named entity recognition (NER) can be performed on the abstracts to extract entities such as genes, chemicals, drugs, and diseases, to create knowledgebase and knowledge graphs. With the advent of generative AI tools, it is possible to further this pipeline to enable a conversational virtual assistant to interact with the full-text articles to reach out to specific and summarized content. Figure 1 shows the schematic representation of the pipeline.

### 3.2 Literature cohort for topic model

Direct queries on PubMed such as “Autism Spectrum Disorder AND multi-omics” or “Autism Spectrum Disorder AND omics” yielded less than 100 articles and did not cover the entire space of different omics studies in ASD. We then created a corpus for topic model from PubMed using a more generic search query “Autism Spectrum Disorder AND *Homo sapiens*” in the last 10 years and used topic modeling to identify articles related to multi-omics studies. The resulting 28,304 abstracts were used for training the topic model.

### 3.3 Choosing the optimal model

Multiple techniques are available to build topic models such as unsupervised, guided, supervised, incremental, multi-modal, and dynamic. As our repertoire consists of a large literature repository, we chose unsupervised (library driven to identify the different research areas covered) and guided (to seed the model to look for specific keywords of our interest). Based on the model coherence metrics, we found that the guided model performed better than unsupervised (for unsupervised model, c_v: 0.61 and u_mass: −4.77). Topic model coherence measures the quality and interpretability of topics generated. We calculated two coherence measures: c_v: based on combination of word co-occurrence, and u_mass: rely solely on document co-occurrence statistics. Furthermore, we fine-tuned the guided model using different parameters (UMAP and HDBSCAN) to select the most optimal model for this cohort. Table 2 shows the performance metrics while fine tuning the guided topic model.

**Table 2:** Performance metrics of different guided topic models.

UMAP param			HDBscan param		Performance		No. of topics
N_neighbors	N_components	Min_dist	Min_cluster_size	Min_samples	C_V	U_mass	
15	2	0.1	100	100	0.438	−5.327	6
15	2	0.1	50	70	0.458	−5.39	6
15	2	0.1	5	50	0.637	−4.47	109
10	2	0.01	100	100	0.652	−3.834	57
10	2	0.01	50	70	0.66	−3.941	93
10	2	0.01	5	50	0.648	−4.328	142
3	2	0.001	100	100	0.6	−4.826	39
3	2	0.001	50	70	0.633	−4.238	89
3	2	0.001	5	50	0.605	−5.043	182
15	5	0	15	None	0.63	−4.57	100
10	2	0.01	5	100	0.61	−4.61	67
10	2	0.01	50	50	0.658	−3.95	112
10	2	0.01	100	50	0.645	−3.87	61
10	2	0.01	50	10	0.64	−4.29	157
10	2	0.01	50	20	0.65	−4.27	134
10	2	0.01	50	30	0.66	−4.2	125
10	2	0.01	50	40	0.669	−3.82	125

We observed that the guided model with 125 topics with “*min_samples*” as 40 showed good coherence (c_v: 0.669 and u_mass: −3.82), and hence, we will be using this model for further discussions. From the input abstracts of 28,304, 10,903 abstracts had a probability score of association to their respective topic greater than 0.8. (Supplementary Table S1). The topics are appended with a numerical value (serial number) for ease of identification.

### 3.4 Multi-omics research trends in ASD

As our focus is on multi-omics studies in ASD, we selected topics associated with multi-omics based on their representative words. Out of the 125 topics, 17 topics were found to be associated with multi-omics-related representative keywords with 1,283 research articles (Table 3). We present our results that show detailed overview of the multi-omics research studies studied in the context of ASD. As shown in Table 2, the multiple omics such as genomics (SNP, mutations and variants, and CNV), transcriptomics (RNA and miRNA), epigenomics (methylation), metabolomics, and microbiomics were classified as individual topics thus spanning the breadth of multi-omics research studies in ASD. Supplementary Figures S1, S2 show the word distribution score and probability distribution of association of abstracts to their multi-omics topics.

**Table 3:** Topics associated with multi-omics that are selected to study the multi-omics research study in ASD.

Name	Representative words	No. of articles
2_synaptic_genes_mice_protein	[‘synaptic’, ‘genes’, ‘mice’, ‘protein’, ‘mouse’, ‘gene’, ‘mutations’, ‘cell’, ‘proteins’, ‘expression’]	160
6_gut_microbiota_microbiome_gi	[‘gut’, ‘microbiota’, ‘microbiome’, ‘gi’, ‘gastrointestinal’, ‘intestinal’, ‘microbial’, ‘axis’, ‘probiotics’, ‘fecal’]	278
15_genes_variants_mutations_novo	[‘genes’, ‘variants’, ‘mutations’, ‘novo’, ‘sequencing’, ‘gene’, ‘genetic’, ‘genome’, ‘rare’, ‘exome’]	53
16_deletion_duplication_16p11_22q11	[‘deletion’, ‘duplication’, ‘16p11’, ‘22q11’, ‘deletions’, ‘syndrome’, ‘region’, ‘microdeletion’, ‘carriers’, ‘duplications’]	51
17_methylation_epigenetic_dna_genes	[‘methylation’, ‘epigenetic’, ‘dna’, ‘genes’, ‘gene’, ‘expression’, ‘chromatin’, ‘serotonin’, ‘histone’, ‘genome’]	103
24_cacna1c_mutation_mutations_timothy	[‘cacna1c’, ‘mutation’, ‘mutations’, ‘timothy’, ‘gene’, ‘syndrome’, ‘qt’, ‘variant’, ‘variants’, ‘ts’]	55
31_genetic_psychiatric_schizophrenia_polygenic	[‘genetic’, ‘psychiatric’, ‘schizophrenia’, ‘polygenic’, ‘adhd’, ‘genome’, ‘disorders’, ‘wide’, ‘bipolar’, ‘major’]	51
38_mirnas_mir_mirna_expression	[‘mirnas’, ‘mir’, ‘mirna’, ‘expression’, ‘rna’, ‘genes’, ‘micrornas’, ‘gene’, ‘cell’, ‘cells’]	56
41_variants_sequencing_exome_variant	[‘variants’, ‘sequencing’, ‘exome’, ‘variant’, ‘pathogenic’, ‘delay’, ‘intellectual’, ‘patients’, ‘disability’, ‘features’]	53
50_gut_microbiota_metabolic_diseases	[‘gut’, ‘microbiota’, ‘metabolic’, ‘diseases’, ‘metabolites’, ‘metabolism’, ‘disease’, ‘microbiome’, ‘carnitine’, ‘mitochondrial’]	57
52_metabolic_metabolism_amino_metabolites	[‘metabolic’, ‘metabolism’, ‘amino’, ‘metabolites’, ‘acid’, ‘acids’, ‘eacute’, ‘aacute’, ‘urine’, ‘plasma’]	51
54_fxs_fragile_fmrp_fmr1	[‘fxs’, ‘fragile’, ‘fmrp’, ‘fmr1’, ‘cgg’, ‘syndrome’, ‘protein’, ‘retardation’, ‘translation’, ‘mrna’]	61
57_snps_association_genetic_polymorphisms	[‘snps’, ‘association’, ‘genetic’, ‘polymorphisms’, ‘genome’, ‘allele’, ‘gene’, ‘haplotype’, ‘wide’, ‘genes’]	50
69_cnvs_cnv_copy_genomic	[‘cnvs’, ‘cnv’, ‘copy’, ‘genomic’, ‘pathogenic’, ‘array’, ‘number’, ‘chromosomal’, ‘variants’, ‘microarray’]	54
96_channel_scn2a_channels_voltage	[‘channel’, ‘scn2a’, ‘channels’, ‘voltage’, ‘variants’, ‘gated’, ‘scn8a’, ‘sodium’, ‘calcium’, ‘mutation’]	50
117_biomarker_biomarkers_asd_mcnvs	[‘biomarker’, ‘biomarkers’, ‘asd’, ‘mcnvs’, ‘therapeutic’, ‘new’, ‘vgcc’, ‘hypothesis’, ‘potential’, ‘nachr’]	50
120_pten_phts_macrocephaly_mutations	[‘pten’, ‘phts’, ‘macrocephaly’, ‘mutations’, ‘hamartoma’, ‘tumor’, ‘germline’, ‘cancer’, ‘mutation’, ‘phosphatase’]	50

**Figure 2:**
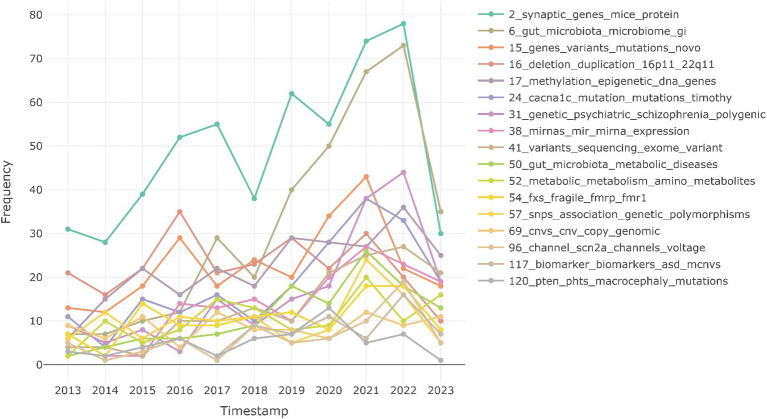
Research trends of different multi-omics topics over time. Timestamps are grouped by year.

Furthermore, topic modeling helps in analyzing the trend of these topics in the last decade. As shown in Figure 2, the trend analysis plot shows that the topic “2_synaptic_genes_mice_protein,” that represents studies associated with synaptic functions, especially mutation studies in mouse models with synaptic genes variants, shows steady increase and is most reported in 2022, followed by studies on impact of gut microbiome on ASD (“6_gut_microbiota_microbiome_gi”). We also observe that studies on *de novo* mutations (“15_genes_variants_mutations_novo”) and 16p and 22q deletions (“16_deletion_duplication_16p11_22q11”) are showing decline in the recent years, while studies associating ASD with PTEN mutations and macrocephaly (“120_pten_phts_macrocephaly_mutations”) and copy number variations (“69_cnvs_cnv_copy_genomic”) are less studied.

**Figure 3:**
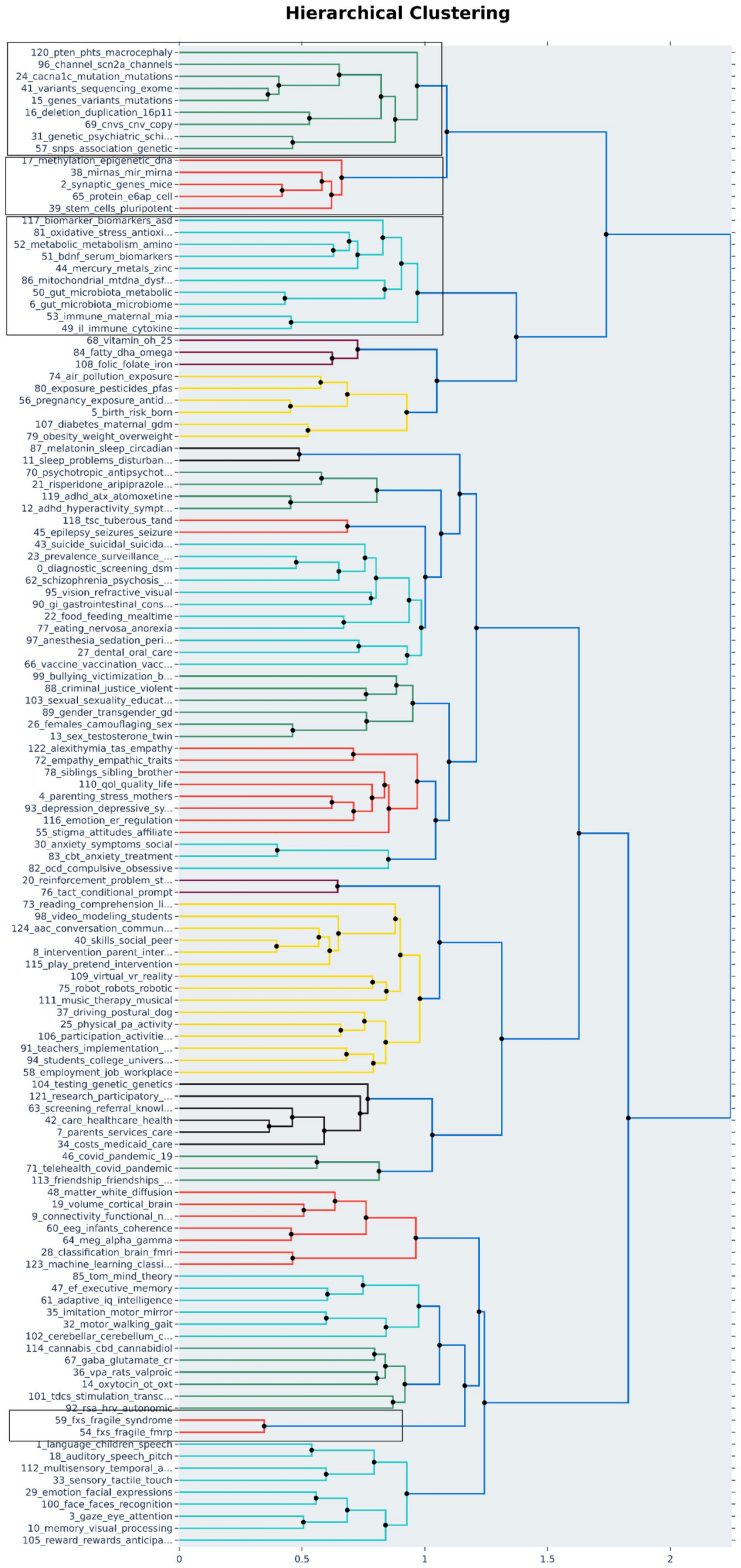
Hierarchical clustering: All topics identified by the topic model are clustered on the basis of distance (Ward’s linkage) to identify the association among the topics. The 125 topics were grouped into 21 clusters out of which 4 clusters contained the multi-omics associated topics. The clusters containing multi-omics topics are highlighted.

We explored the other topics that are closely associated with the above listed multi-omics topics using hierarchical clustering (Figure 3). This further clustering of topics based on distance helps in bringing closely related and studied topics together thus providing directions to analyze a group of topics together. The identified 125 topics are clustered into 21 different clusters out of which four clusters contained one or more shortlisted multi-omics topics shown in Table 3. Out of the 4 clusters, first cluster had only multi-omics-related topics associated with variants, mutations, SNPs, and CNVs. This cluster represents the variant analysis studies in ASD. Furthermore, the second cluster showed 4 multi-omics topics, namely, “50_gut_microbiota_metabolic_diseases,” “6_gut_microbiota_microbiome_gi,” “117_biomarker_biomarkers_asd_mcnvs,” and “52_metabolic_metabolism_amino_metabolites” clustered along with mitochondrial dysfunction, oxidative stress, cytokine-driven immune responses, and metal toxicity related topics. Interestingly, there are publications that are exploring the relationship between gastrointestinal tract (GIT) microbiota and mitochondrial dysfunction in ASD (Hu et al., 2020). This validates the association picked up in hierarchical clustering between multiple topics. The third cluster focuses on synaptic gene studies in mouse models (“2_synaptic_genes_mice_protein”), methylation, and miRNA-associated topics (“17_methylation_epigenetic_dna_genes,” “38_mirnas_mir_mirna_expression”) that are clustered with ubiquitin proteins and stem cell pluripotency topics, highlighting that the mouse models and pluripotent stem cell studies are widely used in studying genetic modifications in ASD (Acab and Muotri, 2015; St. Clair and Johnstone, 2018; Silverman et al., 2022). The fourth cluster is a small, concentrated cluster on fragile X syndrome (FXS) caused due to modifications in the *FMRP* gene (“54_fxs_fragile_fmrp_fmr1”). Thus, clustering of topics can help in deciphering all the related topics in a single view and is useful for screening and selection of studies for further research.

### 3.5 Genes playing key role in ASD

**Figure 4:**
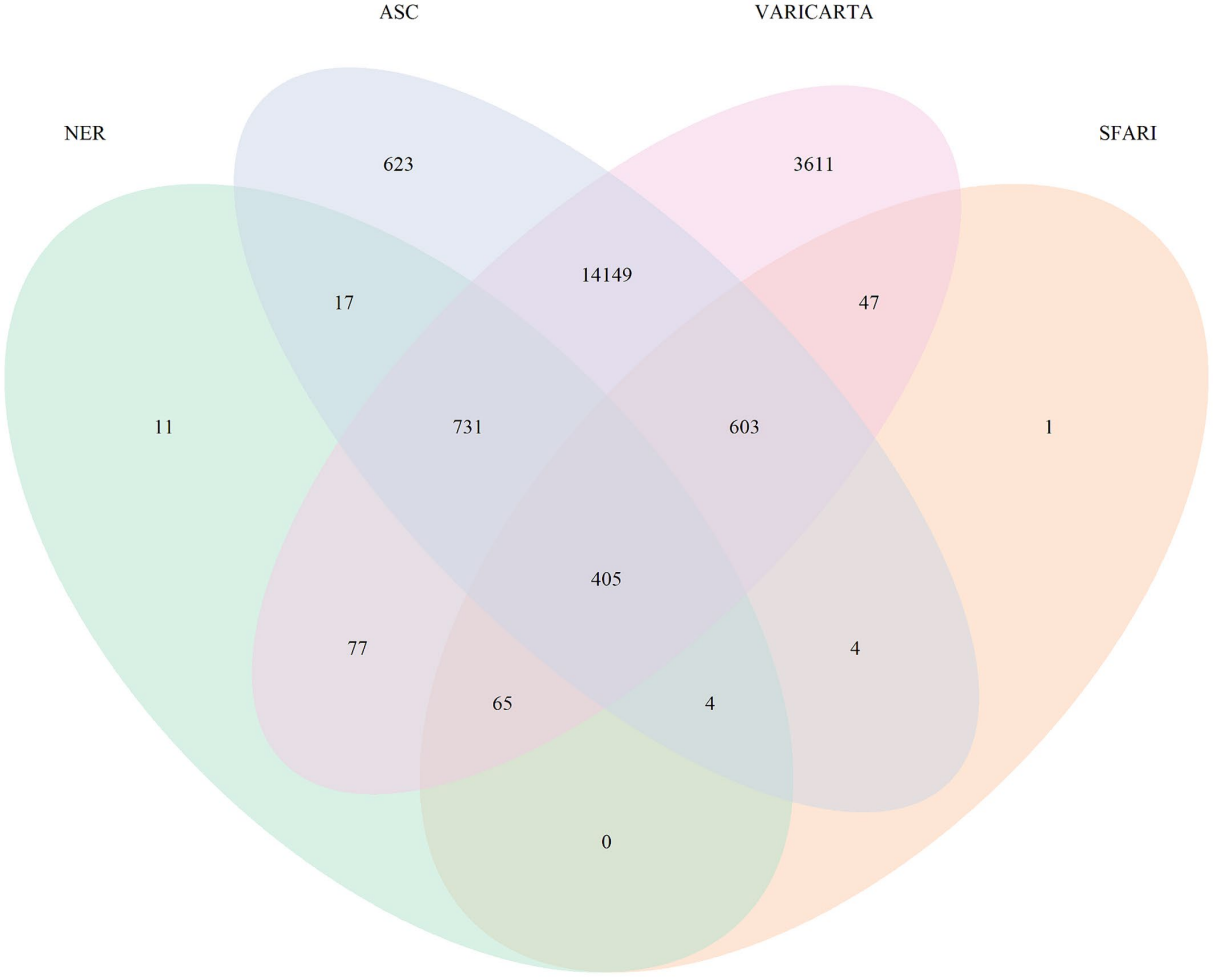
Venn diagram showing the common genes across ASC, SFARI, VariCarta, and our NER list.

We performed named entity recognition (NER) on all the abstracts falling under the 17 multi-omics topics, to identify the key molecular players in ASD (Supplementary Table S2). Of the 17 topics, 10 covered the gene mutations and variants in ASD. We grouped these into a broader category called variants, identified the genes mentioned in these abstracts, and found 1,310 unique gene mentions. For validation, this list of genes was compared with widely used ASD variant databases such as autism spectrum consortia, SFARI, and VariCarta (Abrahams et al., 2013; Belmadani et al., 2019; Satterstrom et al., 2019). Figure 4 shows the common genes across these databases and our gene list from NER. We observe that 11 genes are reported additionally in our list. Upon further manual curation of these 11 genes, we found out that 9 of these genes being explored for their role in ASD and other neurological disorders (Table 4). For instance, one of the genes, *GPRASP2*, variations in this gene are implicated to have a role in autism in females (Butler et al., 2015). There is another study reported in mouse models, to analyze the impact of *GPRASP2* mutations in neurological disorders through knockout experiment (Edfawy et al., 2019). Thus, we showcase that NER method can help in identifying biological entities and their relations and can accelerate the creation of knowledgebase and knowledge graphs. Furthermore, this can highlight research trends and potential gaps to direct future experiments and validate their potential role in ASD.

**Table 4:** Genes identified using NER module of our pipeline that are not reported in ASD variant databases.

Gene Symbol	Gene name	PubMed link	Reported observations in ASD literature
*UBE2A*	Ubiquitin conjugating enzyme E2 A	https://pubmed.ncbi.nlm.nih.gov/23471985/ (Jahanshad et al., 2013)	This gene is related to other neurological disorder
*GPRASP2*	G protein-coupled receptor associated sorting protein 2	https://pubmed.ncbi.nlm.nih.gov/25574603/ (Butler et al., 2015)	The role of this gene is being explored in ASD
*MSNP1*	Moesin pseudogene 1	https://pubmed.ncbi.nlm.nih.gov/27417655/ (Torrico et al., 2017)	This gene has a previously reported GWAS risk variant (could not be replicated)
*AMELY*	Amelogenin Y-linked	https://pubmed.ncbi.nlm.nih.gov/31852540/ (Maxeiner et al., 2019)	This gene is not related to autism
*NAT8B*	N-acetyltransferase 8B	https://pubmed.ncbi.nlm.nih.gov/31945187/ (Rigby et al., 2020)	This gene might play a role dysfunctional ER acetylation which is associated with ASD
*FOXA2*	Forkhead box A2	https://pubmed.ncbi.nlm.nih.gov/32277595/ (Mohammed et al., 2020)	The deletion of this gene might be related to ASD
*TCEAL3*	Transcription elongation factor a like 3	https://pubmed.ncbi.nlm.nih.gov/36368327/ (Hijazi et al., 2022)	This gene is related to other neurological disorder
*MORF4L2*	Mortality factor 4 like 2	https://pubmed.ncbi.nlm.nih.gov/36368327/ (Hijazi et al., 2022)	This gene is related to other neurological disorder
*PEG13*	Paternally expressed 13	https://pubmed.ncbi.nlm.nih.gov/24980697/ (Delgado et al., 2014)	This gene is not yet proven to be associated with ASD
*MTCO2P12*	MT-CO2 pseudogene 12	https://pubmed.ncbi.nlm.nih.gov/25464930/ (Shen et al., 2015)	This gene is reported to play a role in the pathogenesis of autism

We also present the list of genes that have undergone methylation, genes that are reported in microbiome studies and metabolome data in Supplementary Table S2. These genes can potentially be useful in analyzing the molecular patterns in ASD for therapeutic targets and can help in advancing the knowledgebase of ASD.

### 3.6 Generative AI powered summarization

One of the wider used applications of generative AI is its ability to summarize large textual information. In this section, we showcase the ability of generative AI to help in generating concise and collated summaries of topics of interest. We chose topics related to fragile X syndrome (FXS) in autism and found two topics (“54_fxs_fragile_fmrp_fmr1,” “59_fxs_fragile_syndrome_boys”) associated with this syndrome with 116 articles. Using Google cloud services, we used the abstracts to generate summarized content on this topic. Supplementary Table S3 shows the summary of the topics associated with fragile X syndrome reported in the context of ASD. As there are multiple aspects studied in the context of this syndrome, we observe that the summary is divided into behavioral and social aspects in different groups, genetics, psychiatric, etc. Thus, this module helps in generating succinct summaries that can be highly beneficial for report generation.

### 3.7 Generative AI powered conversational chat assistant

**Figure 5:**
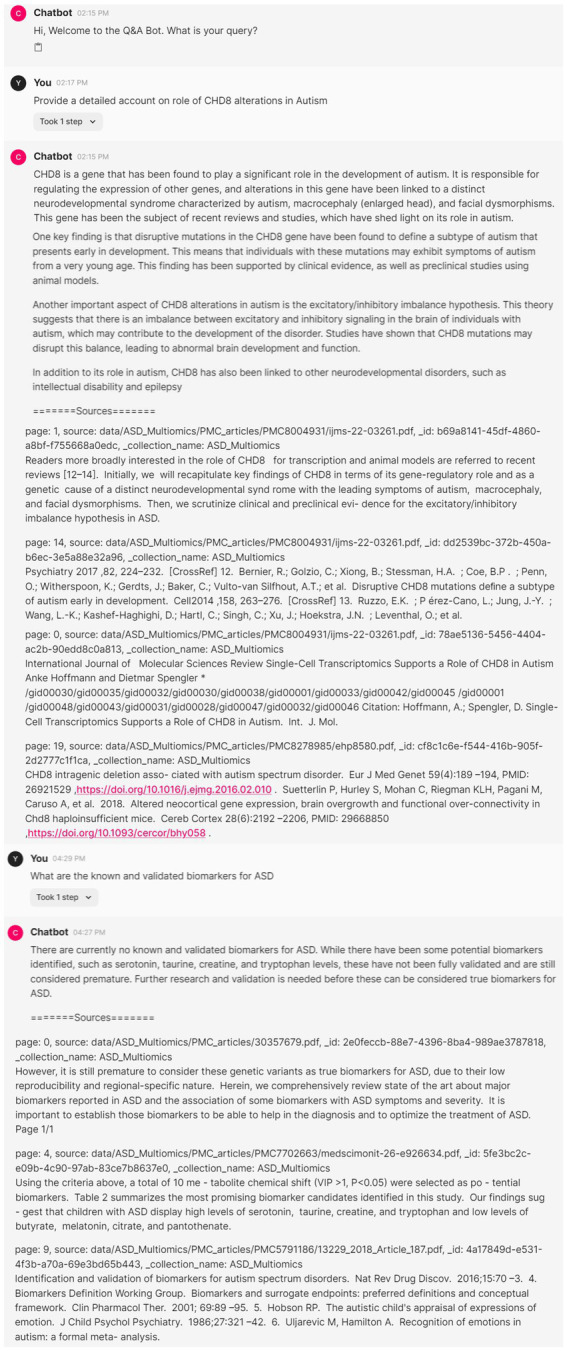
Interactive virtual assistant application to create answers supported with references from scientific literature.

In this section, we highlight the ability to leverage generative AI techniques to build a virtual assistant to quickly mine scientific literature for better insights. We had downloaded all 1,283 multi-omics topic-associated research articles (493 free full-text articles), to enable a chat application that can create an interface to search the articles based on simple linguistic queries. Figure 5 shows the conversation with virtual assistant to reach to specific answers to a given query. The answers are further supported with references for validation, thus, containing the hallucinations of generative AI models. We show that our pipeline significantly reduces the manual efforts required in curating specific information related to a topic.

## 4 Discussion

Literature mining plays a vital role in generating new hypothesis, validation of research, knowledge-gap analysis, and/or to study the market trends. Scientific literature-based evidence gathering has wide applications in pharmaceutical, clinical, and research communities. Tremendous amount of time and manpower is spent to curate and validate valuable information from these studies. We propose a pipeline that can expedite the scoping of data for actionable insights from clinical and biomedical literature. Though we have demonstrated the application using scientific literature, the pipeline can be extended to mine voluminous textual data such as clinical reports and health records.

ASD is a complex disorder which poses difficulty in diagnosis and treatment owing to its heterogenous symptoms. Although there are a few studies that have used multi-omics approaches for studying this disorder (Troisi et al., 2020; Nomura et al., 2021), there is a lack of holistic view of the research studies carried for different omic-profiles of ASD.

Leveraging our pipeline, we provide a bird’s eye view to worm’s eye view of the multi-omics studies in autism in the last 10 years. We have used topic modeling to provide an overview of the different research arenas in ASD. The guided modeling yielded 125 topics or thematic clusters that summarizes the different aspects of ASD studies. We narrowed down the topics by identifying omics-related representative keywords and identified 1,283 research articles that are associated with 17 different multi-omics topics. Gathering these articles directly from search engines would require multiple searches or multiple combinations of keywords. The topics identified showcased high probability of association, thus highlighting the efficiency of the model to mine, identify, and cluster similar articles into a thematic cluster. The identified topic encompassed the genomics, transcriptomics, epigenomics, and microbiomics thus spanning the length and breadth of multi-omics research in ASD. However, topic modeling may not be able to tag all the abstracts into a particular theme, and such abstracts are grouped under “miscellaneous.” These abstracts are not available for further applications of the pipeline. Fine-tuning the modeling with different set of parameters can help in reducing the articles tagged as “miscellaneous.”

Visualizing the results of topic modeling can aid in assessing closely associated topics based on distance, well represented topics, and topic trends over time. This helps in identifying specific topics of interest and their counterparts for downstream actions.

We have highlighted three possible applications in our pipeline. We have used NER to find the genes that have significant genetic modifications such as SNVs, CNVs, and epigenetic changes, which are reported to play a key role in ASD. The identified genes are validated by comparing with public databases on ASD variants, thus emphasizing that our pipeline can facilitate creation of curated knowledge bases.

We have employed the latest generative AI tools and techniques for faster data to insights in the form of a conversational virtual assistant (Q&A) and summarization. Our virtual assistant supports the results with appropriate references thus reducing the hallucinations in the answers and establishes a robust method to validate the answers.

Our literature mining pipeline significantly improves the efforts required in extracting meaningful details from literature. The semi-automated pipeline saves time, cost, and manual efforts required for curation and provides a perfect balance between speed and accuracy. The pipeline is agnostic to domain and can be extended to large cohorts of textual data such as reports, blogs, or any articles. It can address a wide range of applications such as market research, gap analysis, and trend analysis.

## Data availability statement

The original contributions presented in the study are included in the article/Supplementary material, further inquiries can be directed to the corresponding author.

## Author contributions

DM: Writing – review & editing, Conceptualization, Data curation, Formal analysis, Methodology, Software, Visualization. IS: Data curation, Methodology, Writing – original draft, Visualization, Conceptualization, Investigation, Resources, Validation, Supervision, Writing – review & editing. AK: Resources, Validation, Conceptualization, Funding acquisition, Supervision, Writing – review & editing.

## Supplementary material

SUPPLEMENTARY FIGURE S1 Plot showing the word scores of the top 5 words under each topic.

SUPPLEMENTARY FIGURE S2 Plot showing the probability distribution scores of abstracts under each topic.

SUPPLEMENTARY TABLE S1 Results of topic modeling.

SUPPLEMENTARY TABLE S2 Gene lists identified by the NER module from multi-omics abstracts.

SUPPLEMENTARY TABLE S3 Summary of the abstracts tagged under fragile X syndrome (FXS) topic using generative AI tools.
