# Supplementary figures and images for: Deriving comprehensive literature trends on multi-omics analysis studies in autism spectrum disorder using literature mining pipeline
Source: Front Neurosci. 2024 Nov 12;18:1400412. doi: 10.3389/fnins.2024.1400412 (PMC11590066; doi:10.3389/fnins.2024.1400412)

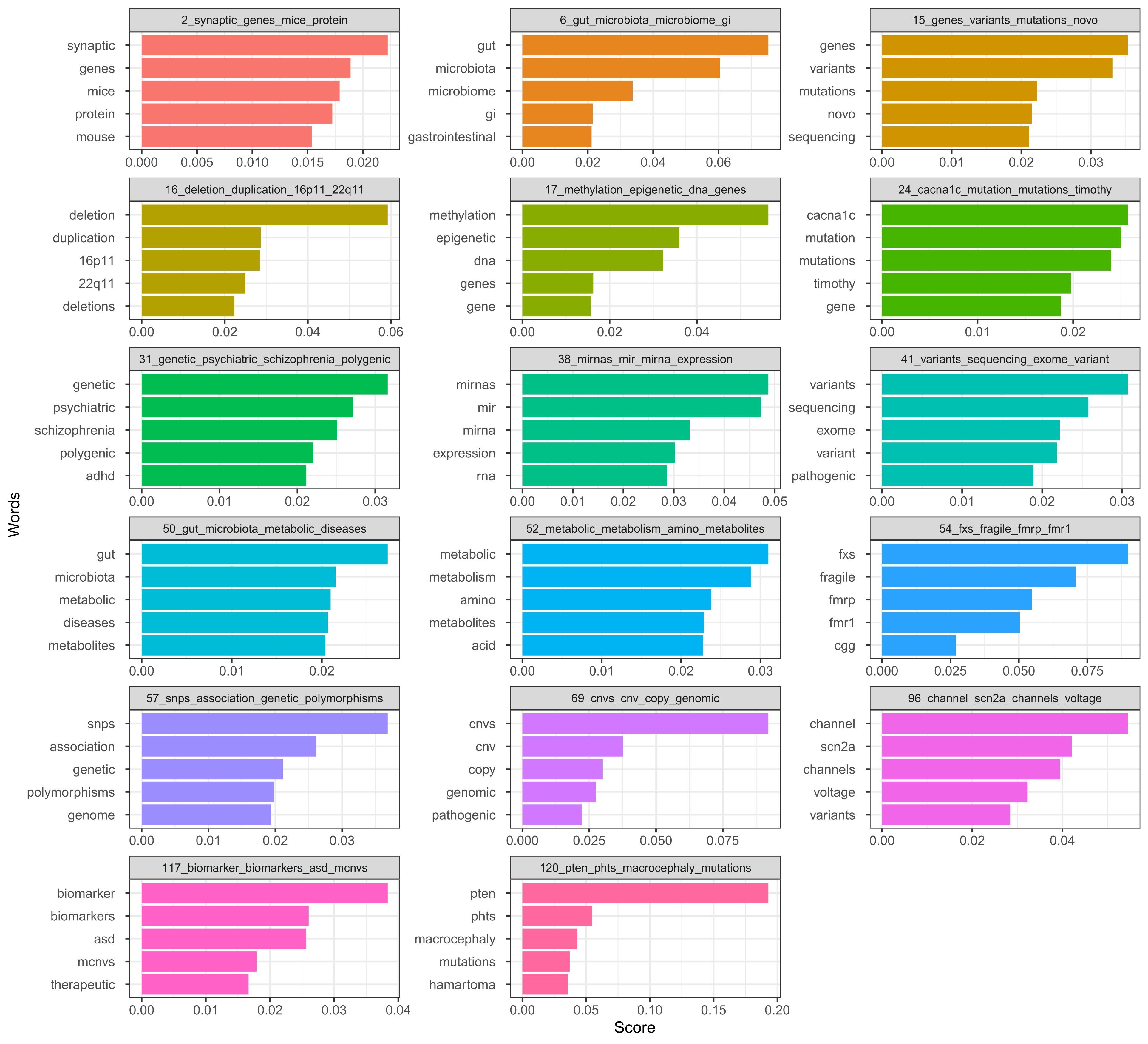

Supplement: SUPPLEMENTARY FIGURE S1 — Plot showing the word scores of the top 5 words under each topic. [file Image_1.JPEG]

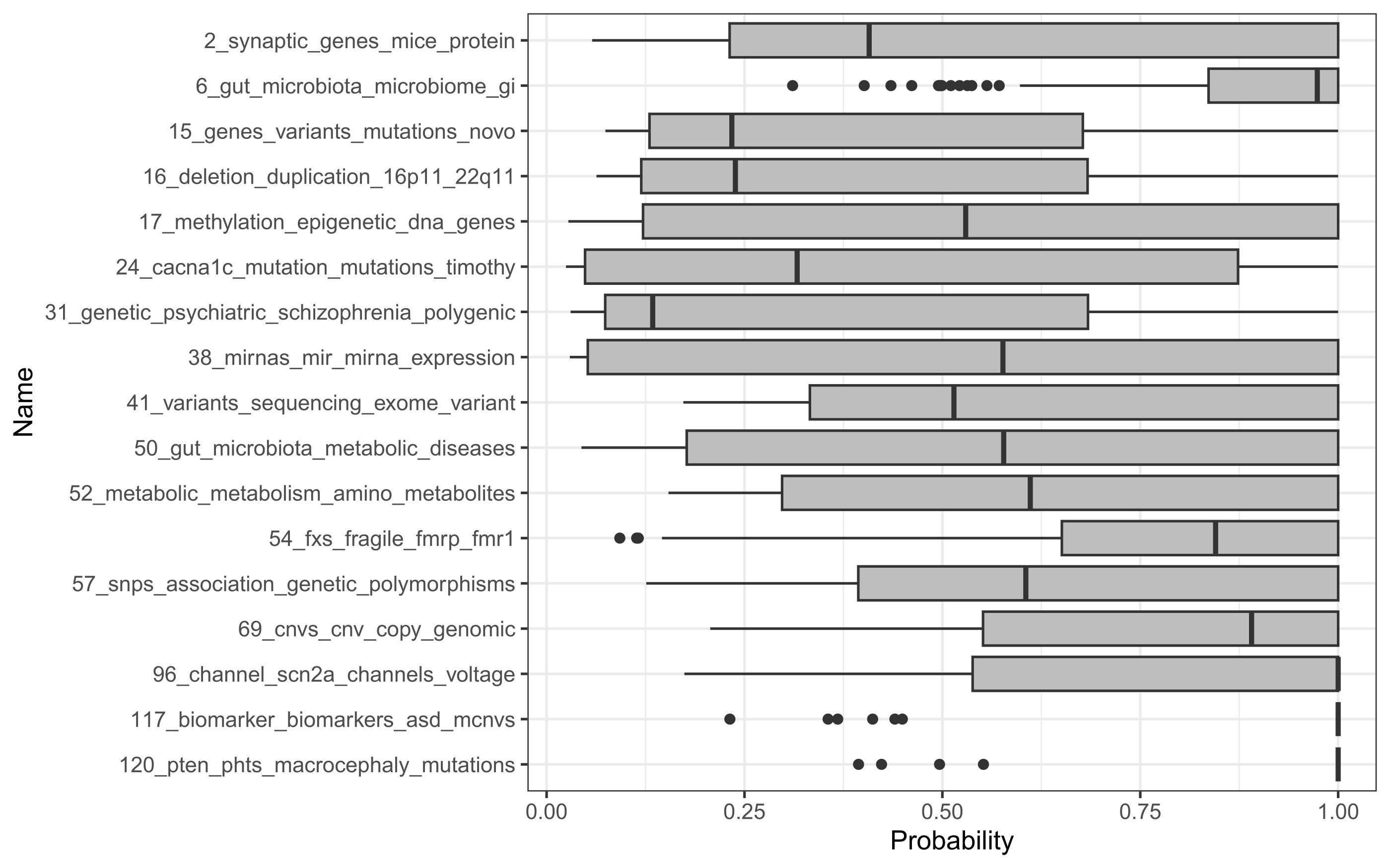

Supplement: SUPPLEMENTARY FIGURE S2 — Plot showing the probability distribution scores of abstracts under each topic. [file Image_2.JPEG]
