# Supplementary material for: Deriving comprehensive literature trends on multi-omics analysis studies in autism spectrum disorder using literature mining pipeline
Source: Front Neurosci. 2024 Nov 12;18:1400412. doi: 10.3389/fnins.2024.1400412 (PMC11590066; doi:10.3389/fnins.2024.1400412)
Supplement: SUPPLEMENTARY TABLE S3 — Summary of the abstracts tagged under fragile X syndrome (FXS) topic using generative AI tools. [file Table_3.DOCX]

**Deriving literature trends and insights on multi-omics analysis studies in Autism Spectrum Disorder using literature mining pipeline**

Dattatray Mongad†, Indhupriya Subramanian†, Anamika Krishanpal*

Life Sciences Research, CTO Organization, Persistent Systems, Pune, India.

† Equal contribution, * Corresponding author

**Supplementary file 3: Summary of the abstracts tagged under fragile X syndrome (FXS) topic using generative AI tools.**

**Behavioral Expression of Autism in Fragile X Syndrome (FXS) Compared to Idiopathic Autism (iAut)**

**Social Impairments**:

- Both FXS+Aut and iAut groups exhibit social impairments, including gaze integration, social overtures, social smile, facial expressions, and response to joint attention.
- However, FXS+Aut individuals score significantly lower (less severe) on these measures, suggesting less severe social impairments.

**Repetitive Behaviors**:

- Rates of stereotypy, self-injury, and sameness behaviors do not differ between groups.
- Compulsive and ritual behavior scores are significantly lower for FXS+Aut compared to iAut.

**Other Behavioral Differences**:

- FXS+Aut individuals show less impairment in Social Smiling than age-, diagnostic-, and severity-matched boys with iAut.
- FXS+Aut individuals show more impairment in complex mannerisms than boys with iAut.

**Longitudinal Trajectories**:

- IQ declines with age in males with FXS+Aut.
- FMRP levels predict change in fluid reasoning, but not in visualization.

**Psychiatric Symptoms**:

- Symptoms of manic/hyperactive behaviors and general anxiety are more frequently reported for boys with FXS than for boys with iAut.
- There is a positive association between social avoidance and general anxiety in FXS that is stronger than that observed in iAut.

**Cognitive and Behavioral Profile**:

- Verbal IQ improves across time in individuals with FXS, while Nonverbal IQ declines.
- Autism ratings decrease; communication and social aspects of adaptive behavior also enhance.
- Elevated levels of hyperactivity/lack of attention at Time 1 significantly improve across three time points, while emotional symptoms, behavioral difficulties, problems with peers, and prosocial behaviors remain stable over time.

**ASD Prevalence and Symptomatology in Females with FXS**:

- Meta-analysis suggests that rates of ASD for females with FXS are reliably higher than for females in the general population (14%).
- Characteristic profiles of impairment are not clear.
- Higher cognitive empathy in girls with FXS may indicate resilience against specific forms of anxiety.

**Daily Living Skills in Males with FXS**:

- Males with FXS need more help/support in domestic and community daily livings skills than in personal daily living skills.
- Reduced daily living skills are associated with lower nonverbal cognition, receptive language, expressive language, and increased autism symptomatology.
- Receptive language emerges as the strongest unique predictor of daily living skill performance.

**Attention-Deficit/Hyperactivity Disorder (ADHD) in Males with FXS**:

- 42% of adolescent and young adult males with FXS meet DSM-5 criteria for ADHD.
- Autism symptom severity and nonverbal cognitive ability do not predict ADHD diagnoses/symptoms.
- DSM-based ADHD-specific scales are recommended over broadband symptom scales to improve accurate identification.

**Social Attention in Preschool Children with FXS and iASD**:

- Children with nsASD display less facial attention than FXS and NT children.
- Lower DQ and elevated ASD severity associate with decreased eye contact in nsASD and FXS.
- Lower DQ is associated with lower social scene attention in FXS.
- Sex, social anxiety, and age are not associated with social attention.

**Repetitive Behaviors in Boys with FXS**:

- Restricted Interests and Sensory Motor behaviors are most problematic.
- Nonverbal IQ is negatively related, whereas anxiety and social affective symptoms of autism spectrum disorder are positively related, to scores for Restricted Interests.
- Anxiety is also positively related to scores for Compulsive behaviors and Ritualistic Sameness behaviors.

**Behavioral Indicators of Social Fear in Preschool Boys with FXS**:

- Boys with elevated ASD symptoms display more avoidant gaze and less facial fear than those with low ASD symptoms across etiologies.
- The iASD group displays more facial fear than the other groups.

**Eye Gaze Avoidance in Boys with FXS and ASD**:

- Boys with FXS obtain significantly higher scores on the Eye Contact Avoidance Scale (ECAS) compared to boys with ASD and MDD.
- Exposure to a brief behavioral treatment probe results in significant decreases in ECAS scores for boys with FXS, but not for boys with ASD.
